# Less Graft-Versus-Host Disease after Rabbit Antithymocyte Globulin Conditioning in Unrelated Bone Marrow Transplantation for Leukemia and Myelodysplasia: Comparison with Matched Related Bone Marrow Transplantation

**DOI:** 10.1371/journal.pone.0107155

**Published:** 2014-09-04

**Authors:** Elias Hallack Atta, Danielli Cristina Muniz de Oliveira, Luis Fernando Bouzas, Márcio Nucci, Eliana Abdelhay

**Affiliations:** 1 CEMO, Instituto Nacional de Câncer, Rio de Janeiro, Brazil; 2 University Hospital, Universidade Federal do Rio de Janeiro, Rio de Janeiro, Brazil; Beth Israel Deaconess Medical Center, Harvard Medical School, United States of America

## Abstract

One of the major drawbacks for unrelated donor (UD) bone marrow transplantation (BMT) is graft-versus-host disease (GVHD). Despite results from randomized trials, antithymocyte globulin (ATG) is not routinely included for GVHD prophylaxis in UD BMT by many centers. One of ways to demonstrate the usefulness of rabbit ATG in UD BMT is to evaluate how its results approximate to those observed in matched related (MRD) BMT. Therefore, we compared the outcomes between UD BMT with rabbit ATG (Thymoglobulin) for GVHD prophylaxis (n = 25) and MRD BMT (n = 91) for leukemia and myelodysplasia. All but one patient received a myeloablative conditioning regimen. Grades II–IV acute GVHD were similar (39.5% vs. 36%, p = 0.83); however, MRD BMT recipients developed more moderate-severe chronic GVHD (36.5% vs. 8.6%, p = 0.01) and GVHD-related deaths (32.5% vs. 5.6%, p = 0.04). UD BMT independently protected against chronic GVHD (hazard ratio 0.23, p = 0.04). The 6-month transplant-related mortality, 1-year relapse incidence, and 5-year survival rates were similar between patients with non-advanced disease in the MRD and UD BMT groups, 13.8% vs. 16.6% (p = 0.50), 20.8% vs. 16.6% (p = 0.37), and 57% vs. 50% (p = 0.67), respectively. Stable full donor chimerism was equally achieved (71.3% vs. 71.4%, p = 1). Incorporation of rabbit ATG in UD BMT promotes less GVHD, without jeopardizing chimerism evolution, and may attain similar survival outcomes as MRD BMT for leukemia and myelodysplasia especially in patients without advanced disease.

## Introduction

Allogeneic hematopoietic stem cell transplantation (HSCT) is the treatment of choice for patients with high-risk hematologic malignancies. However, only 30% of patients have an HLA-matched related donor (MRD) [1]. Unrelated donor (UD) transplantation is an alternative for these patients, particularly with the increasing availability of registered volunteer donors and the regular use of high-resolution HLA typing techniques [2], [3]. However, one of the major drawbacks to UD HSCT is graft-versus-host disease (GVHD), with reported incidences as high as 28% for grades III–IV acute GVHD and 44% for chronic GVHD in patients submitted to an 8/8 allele-level matched UD HSCT [3]. In addition to HLA compatibility, other donor-recipient non-HLA antigen mismatches such as cytokine polymorphisms may also play a role in the development of GVHD [4].

Many approaches have been evaluated to reduce GVHD complications after UD HSCT, especially methods designed to deplete donor alloreactive T cells in the graft [5], [6]. Of particular interest is the administration of antithymocyte globulin (ATG) during the conditioning regimen to promote *in vivo* T cell depletion of the graft [7]–[9]. The use of rabbit ATG as a GVHD prophylaxis agent is more compelling than horse ATG, because of its stronger lymphocytotoxicity secondary to its longer half-life and higher lymphocyte affinity [10], [11]. Three randomized trials demonstrated the efficacy of rabbit ATG in reducing GVHD after UD myeloablative HSCT in patients with hematologic malignancies: two with Thymoglobulin and one with ATG-Fresenius [12]–[14]. Although Thymoglobulin and ATG-Fresenius are rabbit-derived preparations, they differ in the antigen used to elicit the immune response and probably have different biological and clinical properties in HSCT [15], [16].

Only 57% of the European centers routinely include ATG in the prophylaxis for recipients of a graft from an UD, as demonstrated in a survey conducted with 79 out of the 372 centers which perform allogeneic HSCT in Europe [17]. Data from the Center for International Blood and Marrow Transplant Research (CIBMTR) display a similar picture, only 37% of patients with hematologic malignancies submitted to an UD HSCT in 2009 received ATG for GVHD prophylaxis [18]. Therefore, the regular use of ATG in UD HSCT is still controversial, mainly because this approach has not conferred any survival advantage in the randomized trials [19].

One of the ways to assess the role of rabbit ATG in UD HSCT is to evaluate how its outcomes approximate to those observed in MRD HSCT. In the current study, we compared GVHD incidences between MRD bone marrow transplant (BMT) and UD BMT after rabbit ATG (Thymoglobulin) in patients with leukemia and myelodysplasia. Infectious complications and chimerism evolution were also analyzed, since these outcomes might be influenced by the T cell depletion promoted by rabbit ATG in UD BMT. Additionally, survival outcomes were compared to determine whether UD BMT after rabbit ATG achieves similar results as those seen in MRD BMT.

## Patients and Methods

### Eligibility

This study included all consecutive patients submitted to MRD and UD BMT between January 2005 and September 2011 at the Brazilian National Cancer Institute with the following hematologic malignancies: acute lymphoblastic leukemia, acute myelogenous leukemia (AML), chronic myelogenous leukemia, and myelodysplasia. Only patients who received bone marrow as graft were included. This study was approved by the Ethics Committee of the Brazilian National Cancer Institute and was in accordance with the Brazilian legislation and the Declaration of Helsinki. Informed consent was not obtained since the retrospective nature of this study did not affect the healthcare of the included individuals. Moreover, confidentiality was preserved: patient records and data were anonymized and de-identified prior to analysis.

### Conditioning regimens, GVHD prophylaxis, and GVHD treatment

All but one patient underwent a myeloablative conditioning regimen based on either busulfan or total body irradiation (TBI). TBI-based regimens were used mainly in patients with acute lymphoblastic leukemia, and busulfan-based regimens in those with myeloid malignancies. The following regimens were employed: oral busulfan 16 mg/kg and cyclophosphamide 120 mg/kg (n = 63), oral busulfan 16 mg/kg and melphalan 140 mg/m^2^ (n = 2), hyperfractionated TBI 1320 cGy and cyclophosphamide 120 mg/kg (n = 49), hyperfractionated TBI 1320 cGy and melphalan 140 mg/m^2^ (n = 1), or fludarabine 125 mg/m^2^ and melphalan 140 mg/m^2^ (n = 1).

All patients received cyclosporine and methotrexate as GVHD prophylaxis. UD BMT recipients also received rabbit ATG (Thymoglobulin, Sanofi-Aventis); total dose was 8 mg/kg (n = 22), 10 mg/kg (n = 2), or 15 mg/kg (n = 1), beginning on day -4 with its end on day -1. Cyclosporine was adjusted to target a serum level between 200 and 400 ng/mL. Cyclosporine was discontinued after BMT as soon as possible in both the MRD and UD groups, particularly in patients who were GVHD free.

Corticosteroids were used as first-line treatment for grades II–IV acute GVHD, with second-line therapies (extracorporeal photopheresis, anti-tumor necrosis factor antibody, and mycophenolate mofetil) individually or sequentially employed in patients with steroid-refractory acute GVHD. Moderate-severe chronic GVHD was treated with prednisone with the introduction of other agents to unresponsive patients.

### Donor characteristics

All related BMTs were performed with a genotypically identical donor. The UD search process was performed by the Brazilian National Register of Bone Marrow Recipients (REREME). All UDs were matched to the recipient by low/intermediate-resolution technique for HLA-A and HLA-B, and by a high-resolution technique for HLA-DRB1. However, donor-recipient matching with high-resolution techniques for HLA-A, HLA-B, HLA-C, HLA-DQB1, and HLA-DRB1 were also performed in 17 pairs (68% of UD BMT) with only three pairs mismatched at two or more alleles (12% of UD BMT).

### Supportive care and infection prophylaxis

Transplants were performed in single rooms with HEPA filters and positive air pressure. Acyclovir was begun during conditioning and continued until one year post-BMT. Weekly cytomegalovirus (CMV) pp65 antigenemia assays were performed from neutrophil engraftment until day +100 or beyond in cases with extended systemic immunosuppression. Ganciclovir was used for preemptive therapy in patients who developed positive antigenemia. Epstein-Barr virus polymerase chain reaction (PCR) was not routinely performed. Antifungal prophylaxis was prescribed on a case-by-case basis at the discretion of the attending clinician, with the following agents employed: fluconazole (n = 102), voriconazole (n = 12), or lipid-based amphotericin B (n = 2). Granulocyte-colony-stimulating factor (G-CSF) was administered only to patients with life-threatening infections during neutropenia.

### End-points and definitions

The variables analyzed included acute and chronic GVHD, CMV reactivation, CMV disease, invasive fungal disease (IFD), transplant-related mortality (TRM), relapse, overall survival (OS), and chimerism evolution. Infectious complications were analyzed up to day +100, in order to evaluate the impact of rabbit ATG on these outcomes. Acute and chronic GVHD were diagnosed and classified according to the 1994 Consensus Conference and the National Institutes of Health Consensus, respectively [20], [21]. CMV reactivation was defined as the presence of one or more positive cells in a CMV pp65 antigenemia assay, and CMV disease as the combination of symptoms or signs secondary to tissue lesion along with virus detection [22]. Diagnosis of IFD was based on criteria previously established [23]. Chimerism was monitored with PCR-based analysis of variable number of tandem repeats, and more recently with PCR-based analysis of short tandem repeats. Full donor chimerism (FDC) was defined as the establishment of more than 95% donor hematopoietic cells.

Patients were classified into two groups regarding the status of the hematologic malignancy at the time of BMT: advanced disease (acute leukemia beyond second remission or active at BMT, chronic myelogenous leukemia in blast crisis, or myelodysplastic syndrome with excess of blasts) and non-advanced disease (remaining patients).

### Statistical analyses

Baseline characteristics were compared using Chi-square or Fisher’s exact test for categorical variables and Mann-Whitney nonparametric test for continuous variables. Acute and chronic GVHD, IFD, and CMV reactivation were analyzed as time-to-event outcomes with death by other causes treated as a competing event. Patients who did not develop IFD or CMV reactivation until day +100 were censored at that point in time for these outcomes. Patients submitted to donor lymphocyte infusion were also censored at this time for analysis of both acute and chronic GVHD incidences. TRM was measured from BMT until death without previous relapse or progression. Relapse was analyzed as the time from BMT to relapse, with death in remission treated as a competing event. Acute and chronic GVHD, IFD, CMV reactivation, TRM, and relapse were analyzed as cumulative incidence rates with the Fine and Gray’s proportional hazards model to compare outcomes in the presence of competing risks [24]. OS was measured from BMT to death or last follow-up, analyzed with the Kaplan-Meier method, and compared with the log-rank test. Patients submitted to a second HSCT were censored at that time for all outcomes, except for survival analysis. Univariate Cox regression analyses were performed to identify the variables associated with chronic GVHD, variables with a p-value<0.10 were entered in the multivariate model. All p-values were two-sided, with p<0.05 indicating statistical significance. Registration and analysis of data were carried out using IBM SPSS version 15 software. Cumulative incidences, the Fine and Gray’s test, and graphs were generated using R Statistical Software (R-project version 3.0.1, http://www.r-project.org/).

## Results

### Comparison of baseline characteristics

During the study period, 91 MRD and 25 UD BMT were identified and analyzed. Baseline characteristics were similar between the groups, except that the UD BMT group was more likely to have advanced disease at BMT, receive a graft with donor-recipient ABO mismatch, and be submitted to a TBI-based conditioning regimen than the MRD BMT group (Table 1).

**Table 1:** Comparison of baseline characteristics between matched related and unrelated BMT.

	Matched related BMT (n = 91)	Unrelated BMT (n = 25)	p value
Age (years), median (range)	31 (3–64)	17 (4–55)	0.11
Male gender, n (%)	59 (64.8%)	15 (60%)	0.65
Diagnosis, n (%)			
ALL	26 (28.6%)	12 (48%)	0.09
AML	31 (34.1%)	5 (20%)	0.22
MDS	13 (14.3%)	5 (20%)	0.53
CML	21 (23%)	3 (12%)	0.27
Advanced disease at the time of BMT, n (%)	19 (20.9%)	13 (52%)	0.004
Previous HSCT, n (%)	5 (5.5%)	2 (8%)	0.64
Pre-BMT positive CMV serology, n (%)	84 (92.3%)	22 (88%)	0.44
Serum ferritin before BMT (mg/dL), median (range)	881 (8.5–7496)	1367 (18.9–10474)	0.16
Donor-recipient gender mismatch, n (%)	39 (42.9%)	15 (60%)	0.17
ABO match	68 (74.7%)	8 (32%)	0.0002
TBI-based preparative regimen, n (%)	31 (34.1%)	19 (76%)	0.0002
Busulfan-based preparative regimen, n (%)	59 (64.8%)	6 (24%)	0.0005
Rabbit ATG dose (mg/kg), median (range)	0	8 (8–15)	<0.0001
Nucleated cell dose (x10^8^/Kg), median (range)	2.39 (1.01–6.04)	2.92 (1.22–8.50)	0.50
G-CSF before day +15, n (%)	5 (5.5%)	2 (8%)	0.64

Abbreviations: ALL, acute lymphoblastic leukemia; AML, acute myelogenous leukemia; ATG, antithymocyte globulin; BMT, bone marrow transplantation; CML, chronic myelogenous leukemia; CMV, cytomegalovirus; G-CSF, granulocyte colony-stimulating factor; HSCT, hematopoietic stem cell transplantation; MDS, myelodysplastic syndrome; TBI, total body irradiation.

### Graft-versus-host disease

**Figure 1:**
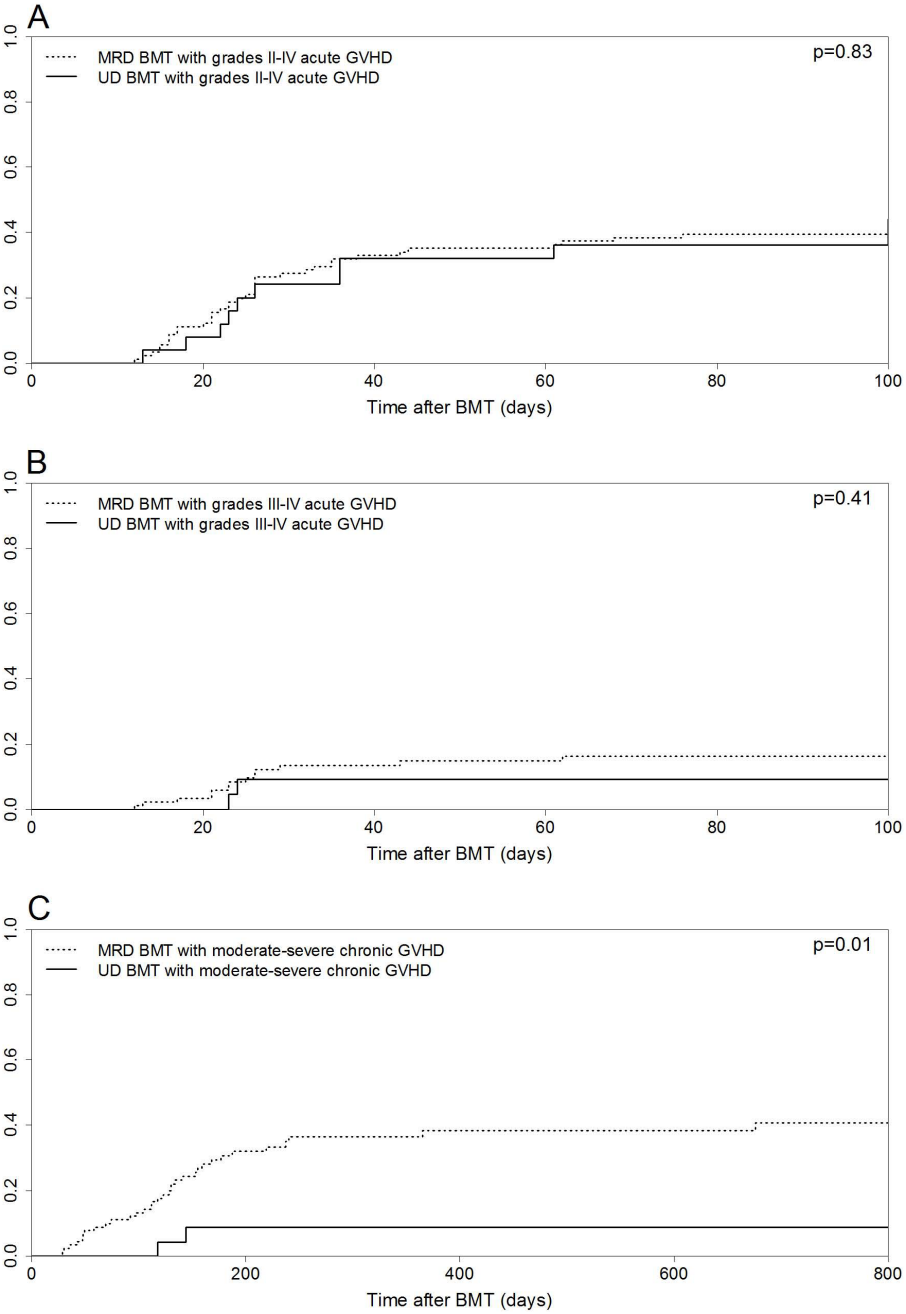
Cumulative incidence curves for matched related donor (MRD) and unrelated donor (UD) bone marrow transplantation (BMT). (A) Cumulative incidences for grades II–IV acute graft-versus-host disease (GVHD). (B) Cumulative incidences for grades III–IV acute GVHD. (C) Cumulative incidences for moderate-severe chronic GVHD.

The cumulative incidence of grades II–IV acute GVHD at day +100 was 39.5% (95% confidence interval [CI] 29.4–49.4%) and 36% (95% CI 17.7–54.6%) in patients submitted to MRD and UD BMT, respectively (p = 0.83, Figure 1A). Grades III–IV acute GVHD incidences at day +100 were also similar: 16.2% (95% CI 9–25.2%) for MRD and 9.1% (95% CI 1.4–25.6%) for UD BMT (p = 0.41, Figure 1B). Steroid-refractory acute GVHD was observed in 31.4% (11 out of 35) and 20% (two out of 10) of patients who developed acute GVHD in the MRD and UD BMT groups, respectively (p = 0.69).

The 1-year incidence of moderate-severe chronic GVHD was higher in MRD than in UD BMT recipients: 36.5% (95% CI 26.1–46.9%) vs. 8.6% (95% CI 1.3–24.6%, p = 0.01, Figure 1C). Multivariate analysis indicated that UD BMT remained independently associated with moderate-severe chronic GVHD, displaying a protective effect (hazard ratio [HR] 0.23, 95% CI 0.05–0.95, p = 0.04, Table 2).

**Table 2:** Cox regression analysis of variables predictive for moderate-severe chronic GVHD.

	Univariate analysis		Multivariate analysis	
	p value	Hazard ratio (95% CI)	p value	Hazard ratio (95% CI)
Age	0.03	1.02 (1.00–1.03)	0.05	–
ALL	0.45	0.75 (0.36–1.58)	–	–
AML	0.81	1.09 (0.52–2.27)	–	–
MDS	0.13	0.40 (0.12–1.33)	–	–
CML	0.03	2.09 (1.03–4.20)	0.06	–
Advanced disease at BMT	0.22	0.55 (0.21–1.43)	–	–
Pre-BMT serum ferritin	0.73	1.00 (1.00–1.00)	–	–
Donor-recipient gender mismatch	0.12	1.69 (0.87–3.29)	–	–
Busulfan-based conditioning	0.35	1.38 (0.69–2.75)	–	–
TBI-based conditioning	0.35	0.72 (0.36–1.43)	–	–
Unrelated BMT	0.04	0.23 (0.05–0.95)	0.04	0.23 (0.05–0.95)
Total nucleated cell dose	0.13	0.78 (0.57–1.07)	–	–

Abbreviations: ALL, acute lymphoblastic leukemia; AML, acute myelogenous leukemia; BMT, bone marrow transplantation; CML, chronic myelogenous leukemia; CI, confidence interval; GVHD, graft-versus-host disease; MDS, myelodysplastic syndrome; TBI, total body irradiation.

### Infectious complications

**Figure 2:**
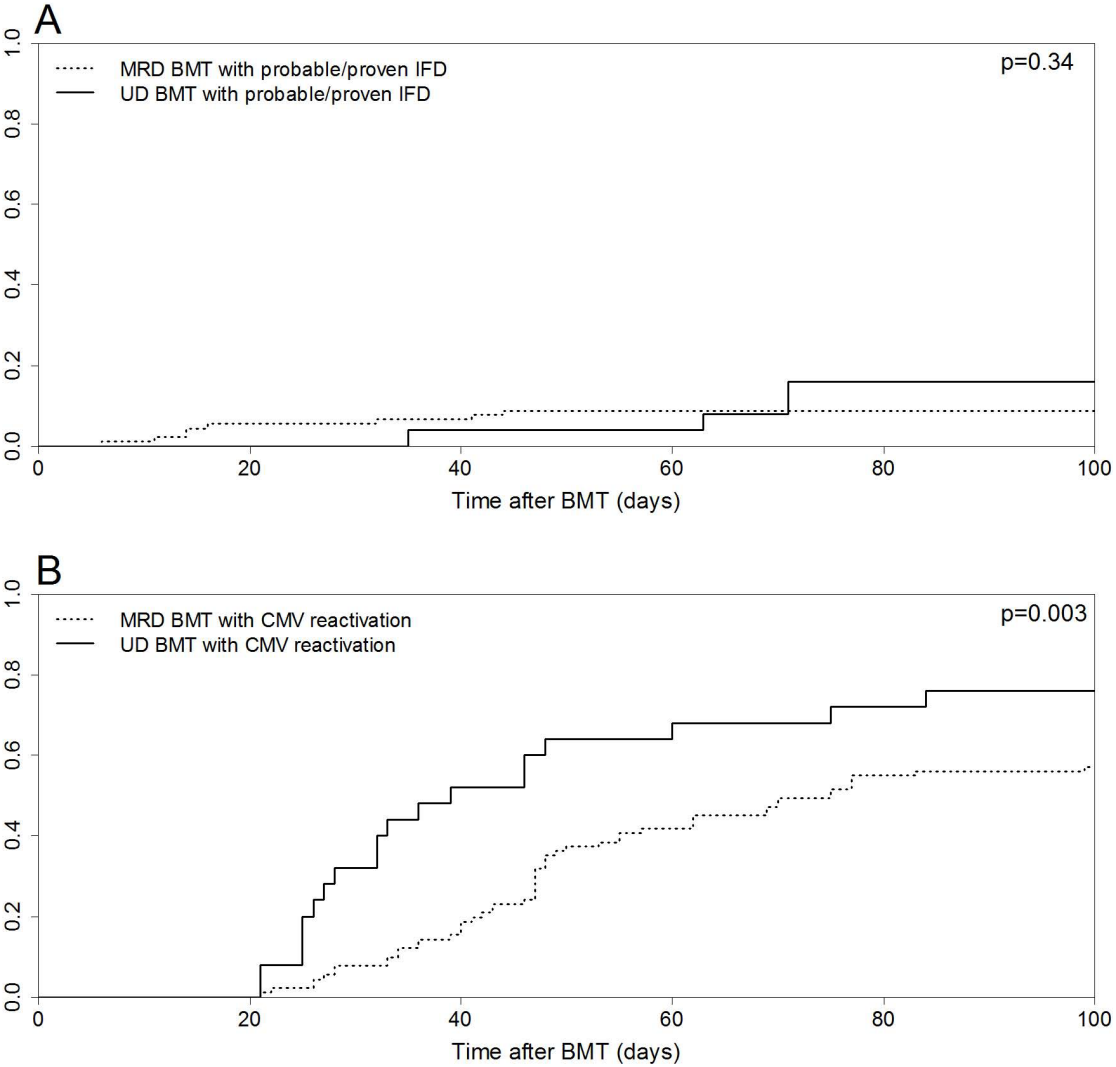
Cumulative incidence curves for matched related donor (MRD) and unrelated donor (UD) bone marrow transplantation (BMT). (A) Day +100 probability for proven or probable invasive fungal disease (IFD). (B) Day +100 probability for cytomegalovirus (CMV) reactivation.

The cumulative incidences of proven or probable IFD on day +100 were 8.7% (95% CI 4–15.7%) and 16% (95% CI 4.8–33%) in MRD and UD BMT recipients (p = 0.34, Figure 2A). Eight cases of proven/probable IFD developed in MRD BMT patients: invasive aspergillosis (n = 4) and invasive candidiasis (n = 4); whereas the UD BMT group registered four cases: invasive aspergillosis (n = 2), disseminated fusariosis (n = 1), and cryptococcosis (n = 1).

CMV reactivation incidence at day +100 was higher in UD than in MRD BMT: 76% (95% CI 52–89.1%) vs. 57.1% (95% CI 46.2–66.6%, p = 0.003, Figure 2B). However, only 6.6% of the MRD (six out of 91) and 8% of the UD BMT (two out of 25) patients developed CMV disease (p = 0.68).

### Transplant-related mortality, relapse, and survival

The median follow-up for all patients was 592.5 days (range, 12–2948 days). Since there were more patients with advanced disease in the UD BMT group, the results regarding TRM, relapse, and OS were classified into two categories: non-advanced and advanced disease.

**Figure 3:**
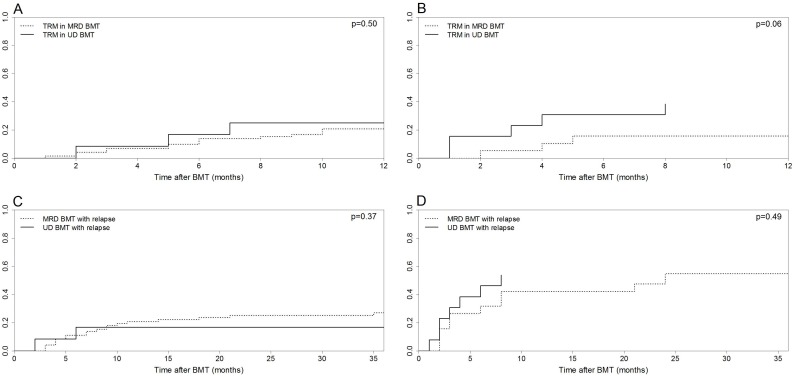
Cumulative incidence curves for matched related donor (MRD) and unrelated donor (UD) bone marrow transplantation (BMT). (A) Transplant-related mortality (TRM) in patients with non-advanced disease at the time of BMT. (B) TRM in patients with advanced disease at the time of BMT. (C) Relapse incidences in patients with non-advanced disease at the time of BMT. (D) Relapse incidences in patients with advanced disease at the time of BMT.

In patients with non-advanced disease at the time of BMT, the 6-month TRM was similar between the two groups, 13.8% (95% CI 7–22.9%) in MRD and 16.6% in UD BMT (95% CI 2.3–42.5%, p = 0.50, Figure 3A). However, in patients with advanced disease, the 6-month TRM was higher in UD BMT: 30.7% (95% CI 8.4–56.9%) vs. 15.7% (95% CI 3.6–35.7%, p = 0.06, Figure 3B). The high mortality observed in patients with advanced disease receiving UD BMT was mainly attributable to infectious complications: CMV disease (n = 2), disseminated fusariosis (n = 1), neurotoxoplasmosis (n = 1), and *Pseudomonas aeruginosa* bacteremia (n = 1).

The 1-year relapse incidences were similar between the MRD and UD BMT groups both among patients with non-advanced disease (20.8% [95% CI 12.3–30.9%] and 16.6% [95% CI 22.9–42.7%], respectively, p = 0.37, Figure 3C) and advanced disease (42.1% [95% CI 19.5–63.2%] and 53.8% [95% CI 21.7–77.8%], respectively, p = 0.49, Figure 3D).

**Figure 4:**
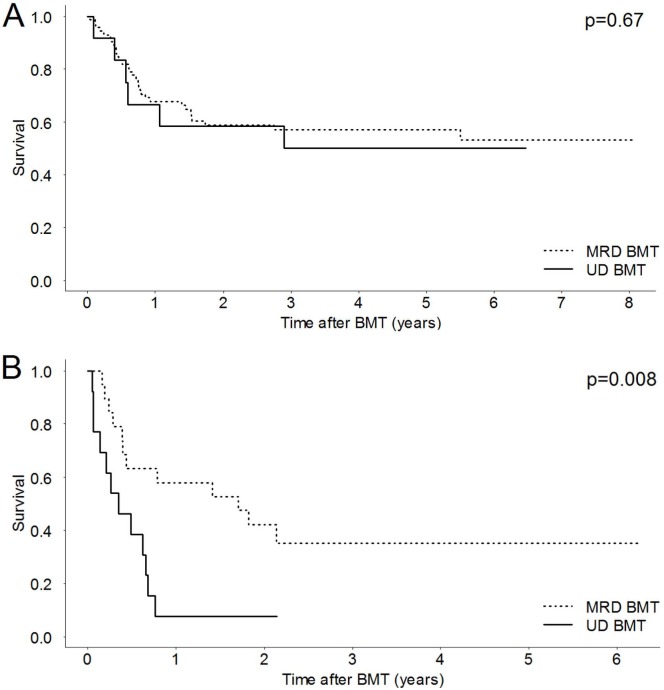
Kaplan-Meier curves for matched related donor (MRD) and unrelated donor (UD) bone marrow transplantation (BMT). (A) Overall survival in patients with non-advanced disease at the time of BMT. (B) Overall survival in patients with advanced disease at the time of BMT.

No difference was found in the 5-year OS between patients with non-advanced disease submitted to MRD and UD BMT: 57% and 50%, respectively (p = 0.67, Figure 4A). However, the 5-year OS was higher in MRD BMT patients with advanced disease: 35.1% vs. 7.7% (p = 0.008, Figure 4B).

Relapse of the hematologic malignancy was the most common cause of death in the population analyzed (Table 3). However, GVHD-related deaths were more common in MRD than in UD BMT recipients, 32.5% (14 out of 43) vs. 5.6% (one out of 18), p = 0.04. On the contrary, infection-related deaths were less common in MRD than in UD BMT recipients, 4.7% (two out of 43) vs. 33.3% (six out of 18), p = 0.006.

**Table 3:** Comparison of death causes between related and unrelated BMT.

Death causes	Related BMT (n = 91)	Unrelated BMT (n = 25)	p value
Relapse, n (%)	23 (53.4%)	8 (44.4%)	0.58
Infection, n (%)	2 (4.7%)	6 (33.3%)	0.006
Toxicity, n (%)	2 (4.7%)	2 (11.1%)	0.57
GVHD, n (%)	14 (32.5%)	1 (5.6%)	0.04
Other, n (%)	2 (4.7%)	1 (5.6%)	1.00

Abbreviations: BMT, bone marrow transplantation; GVHD, graft-versus-host disease.

### Chimerism data

Chimerism assessment was available in 88.9% (80 out of 90) and 95.5% (21 out of 22) of patients surviving beyond day +30 in the MRD and UD BMT groups, respectively (p = 0.68). The proportion of patients who achieved stable FDC was similar between the groups, 71.3% in MRD and 71.4% in UD BMT recipients (p = 1).

## Discussion

Our results suggest that the incorporation of rabbit ATG in UD BMT protects against GVHD-related complications, with less chronic GVHD and fewer GVHD-related deaths in UD compared to MRD BMT. In addition, UD BMT after rabbit ATG was independently associated with protection against chronic GVHD. Despite the observed efficacy of rabbit ATG for GVHD prophylaxis in UD BMT, achievement of stable FDC was not impaired. TRM, relapse, and OS were similar between MRD and UD BMT in patients with leukemia and myelodysplasia without advanced disease at the time of BMT, in the setting where most patients received a myeloablative conditioning regimen.

The incidence of grades II–IV acute GVHD in our UD BMT recipients was 40.9%, with only 10% developing grades III–IV acute GVHD. These incidences are lower than those reported in two randomized trials conducted by Bacigalupo *et al*, in which grades II–IV acute GVHD developed in 72% and 79% of patients and grades III–IV developed in 36% and 50% of patients in the UD BMT arms without rabbit ATG (Thymoglobulin) [12]. In another study with the National Marrow Donor Program data, the incidences of grades III–IV acute GVHD were 28%, 37%, and 44% in patients undergoing an 8/8 (n = 1,840), 7/8 (n = 985), and 6/8 (n = 633) allele-matched UD HSCT, respectively; incidences substantially higher than that observed in our study [3]. Therefore, rabbit ATG protected our UD BMT recipients against acute GVHD; reducing its incidence to that observed in MRD BMT. Moreover, a smaller proportion of the UD BMT recipients developed severe (grades III–IV) and steroid-refractory acute GVHD; however, these differences were not statistically significant possibly due to the small number of cases. In our study, moderate-severe chronic GVHD was considerably more common after MRD than after UD BMT. In addition, UD BMT with rabbit ATG was independently associated with protection against chronic GVHD by multivariate analysis. This protective effect was observed despite some patients who were not matched by high-resolution techniques for HLA class I antigens or not completely HLA allele-matched with their UD. Previous studies have also observed an inferior incidence of chronic GVHD in recipients of UD HSCT conditioned with rabbit ATG (Thymoglobulin, total dose ≥6 mg/kg) compared to MRD HSCT, without differences in acute GVHD incidences [25], [26]. However, differently from these studies, our population was uniform with respect to stem cell source, since all patients received bone marrow as graft [25], [26]. Another important finding from our study was that, despite protecting against GVHD, conditioning with rabbit ATG did not jeopardized the achievement of stable FDC in UD BMT recipients. To our knowledge, the current study is the first to demonstrate that, at least in the setting where most patients received a myeloablative regimen, MRD and UD BMT after rabbit ATG (Thymoglobulin) display a similar chimerism profile.

Infection-related deaths were the main cause of non-relapse mortality in UD BMT recipients, especially in those with advanced disease. Infectious complications were manageable in UD BMT patients without advanced disease, owing to adequate prophylaxis and treatment. Despite a higher incidence of CMV reactivation in UD BMT, this was not translated to more cases of CMV disease, highlighting the efficacy of the preemptive approach adopted. IFD incidence was slightly higher in the UD BMT group, although this difference was not statistically significant. No case of post-transplant lymphoproliferative disorder was observed; however, our UD BMT population was not large, and the regular monitoring of Epstein-Barr virus DNA levels after rabbit ATG conditioning is recommended to allow preemptive treatment. Most of our UD BMT patients received rabbit ATG (Thymoglobulin) at a total dose of 8 mg/kg, a dose which probably allows a balance between protection against GVHD and an increase in the risk of infectious complications [27], [28]. A study conducted by Remberger *et al* found that the ideal dose of Thymoglobulin for GVHD prophylaxis in UD myeloablative HSCT is 6–8 mg/kg, since lower doses (4 mg/kg) increased the risk of severe acute GVHD, whereas higher doses (10 mg/kg) promoted more infectious death [28]. In reduced-intensity HSCT, the optimal dose of Thymoglobulin was 6 mg/kg, since an 8 mg/kg dose was associated with an inferior relapse-free survival particularly in patients with high-risk disease [29].

Patients with non-advanced disease undergoing UD BMT experienced similar TRM, relapse, and OS compared with MRD BMT recipients. However, TRM was higher in UD BMT recipients with advanced disease, promoting poor survival in this subgroup. This higher TRM was probably secondary to the use of fully myeloablative regimens in patients with low functional reserve after multiple lines of chemotherapy to control disease progression during the UD search. Previous studies in patients with hematologic malignancies also found similar TRM, relapse, and OS between UD myeloablative HSCT after rabbit ATG (Thymoglobulin) and MRD HSCT [25], [26], [30]. However, a recent retrospective analysis with data from the Center for International Blood and Marrow Transplant Research (CIBMTR) found that ATG conditioning in reduced-intensity HSCT increased the likelihood of relapse, which negatively influenced disease-free survival in patients with hematologic malignancies [31]. Therefore, immune modulation with ATG in the reduced-intensity setting might abrogate the graft-versus-leukemia effect that is responsible for the elimination of residual malignant cells that persist after a less intensive preparative regimen.

Our study has various limitations. First, data was retrospectively collected; however, information was easily retrieved with minimal loss. Second, some baseline characteristics were different between the groups and this could be associated with the observed outcomes. For instance, the UD group was younger and composed mainly by patients with acute lymphoblastic leukemia. However, by multivariate analysis, we were able to identify the variables independently associated with chronic GVHD. Another limitation is the small and heterogeneous patient population, particularly in the UD BMT group. The small sample size probably precluded the detection of other clinically significant differences between the groups and also hampered multivariate Cox regression analyses regarding outcomes such as relapse and OS. Finally, to counteract the higher mortality observed in the UD BMT group, chronic GVHD cumulative incidence was estimated using death without this event as a competing risk.

Notwithstanding these limitations, the current study has implications for both the research and clinical settings as several outstanding questions remain. In the translational research setting, more research is needed to understand the exact mechanisms by which rabbit ATG prevents GVHD: Which specific cells from the allograft are depleted and what are rabbit ATG’s effects on the recipient [32], [33]. From a clinical standpoint, our study suggests that UD HSCT after myeloablative conditioning with rabbit ATG offers less GVHD with similar survival outcomes as MRD HSCT for leukemia and myelodysplasia, especially in patients without advanced disease at BMT. These results are particularly important because they differ from the current transplant strategy adopted by many centers as demonstrated in two recent studies with CIBMTR data which also compared UD and MRD HSCT for AML (8/8 UD, n = 1193; MRD, n = 624) and myelodysplasia (8/8 UD, n = 413; MRD, n = 176) [34], [35]. Despite similar survival rates, B–D acute GVHD was more common after 8/8 allele-matched UD than MRD HSCT both for AML (51% vs. 33%, p<0.001) and myelodysplasia (54% vs. 42%, p = 0.006). Also, the 1-year probability of chronic GVHD was higher in AML patients submitted to an 8/8 allele-matched UD HSCT (45% vs. 39%, p = 0.008). In the previously cited studies, the higher incidence of GVHD was mainly because most patients were not conditioned with ATG and received peripheral blood as graft. However, ATG conditioning might also counteract the increased risk of GVHD after peripheral blood HSCT [36]. The burden of GVHD should be taken into account in UD HSCT, because it might increase TRM, worsen quality of life, prolong immunosuppressive therapy, and increase health care costs [13], [36], [37]. Both acute and chronic GVHD are associated with worse quality of life in many aspects: physical functioning, role functioning, social functioning, mental health, and general health [38]. Therefore, although UD HSCT might be the only option for patients without a MRD, physicians may offer improved quality of life through GVHD reduction [39]. Finally, our results were obtained after rabbit ATG (Thymoglobulin) conditioning; therefore, comparative studies are necessary to characterize the differences among ATG brands and other *in vivo* T-cell depleting agents in UD HSCT.

In summary, our results demonstrate the protective effect of rabbit ATG against GVHD in UD BMT, overcoming donor-recipient HLA and non-HLA disparities. Incorporation of rabbit ATG in the preparative regimen of UD BMT promotes less GVHD compared to MRD BMT, without jeopardizing the achievement of stable FDC, and may attain similar TRM, relapse, and survival in patients with leukemia and myelodysplasia especially in those without advanced disease at BMT. Prospective randomized trials in the era of high-resolution HLA typing techniques and better supportive care are needed to define the exact role of rabbit ATG in UD HSCT.
